# Clonal Differences between Non-Typhoidal *Salmonella* (NTS) Recovered from Children and Animals Living in Close Contact in The Gambia

**DOI:** 10.1371/journal.pntd.0001148

**Published:** 2011-05-31

**Authors:** Michel M. Dione, Usman N. Ikumapayi, Debasish Saha, Nuredin I. Mohammed, Stanny Geerts, Margareta Ieven, Richard A. Adegbola, Martin Antonio

**Affiliations:** 1 International Trypanotolerance Centre, Banjul, The Gambia; 2 Medical Research Council (UK) Laboratories, Banjul, The Gambia; 3 Institute of Tropical Medicine, Antwerp, Belgium; 4 University of Antwerp, Antwerp, Belgium; Michigan State University, United States of America

## Abstract

**Background:**

Non-Typhoidal *Salmonella* (NTS) is an important cause of invasive bacterial disease and associated with mortality in Africa. However, little is known about the environmental reservoirs and predominant modes of transmission. Our study aimed to study the role of domestic animals in the transmission of NTS to humans in rural area of The Gambia.

**Methodology:**

Human NTS isolates were obtained through an active population-based case-control surveillance study designated to determine the aetiology and epidemiology of enteric infections covering 27,567 Gambian children less than five years of age in the surveillance area. Fourteen children infected with NTS were traced back to their family compounds and anal swabs collected from 210 domestic animals present in their households. Identified NTSs were serotyped and genotyped by multi-locus sequencing typing.

**Principal Findings:**

NTS was identified from 21/210 animal sources in the households of the 14 infected children. Chickens carried NTS more frequently than sheep and goats; 66.6%, 28.6% and 4.8% respectively. The most common NTS serovars were *S*. Colindale in humans (21.42%) and *S*. Poona in animals (14.28%). MLST on the 35 NTS revealed four new alleles and 24 sequence types (ST) of which 18 (75%) STs were novel. There was no overlap in serovars or genotypes of NTS recovered from humans or animal sources in the same household.

**Conclusion:**

Our results do not support the hypothesis that humans and animals in close contact in the same household carry genotypically similar *Salmonella* serovars. These findings form an important baseline for future studies of transmission of NTS in humans and animals in Africa.

## Author Summary

Salmonellosis is a neglected tropical disease causing serious dysentery and septicaemia particularly in young infants, elderly and immunocompromised individuals such as HIV patients and associated with substantial mortality in developing countries. Salmonellosis also constitutes a major public health problem as it is considered the most widespread bacterial zoonosis of food origin throughout the world. Many epidemiological data exist from developed countries concerning transmission of Non-Typhoidal *Salmonella* (NTS) but few are available from developing countries. In addition few studies in sub-Saharan Africa have considered the interface between humans and their environment in relation to animals present in the household and food hygiene. This study describes the prevalence of NTS among fourteen Gambian children and 210 domestic animals living in close proximity (household) to the children in a rural setting in The Gambia. We found that the domestic animals living in the same household as patients carried different NTS serovar and genotypes; indicating that zoonotic transmission does not occur in our setting. This study provides baseline data for future studies of transmission of NTS in rural Africa.

## Introduction

Non-Typhoidal *Salmonella* (NTS) are important causes of invasive bacterial diseases and are associated with substantial mortality. In rural Gambia, NTS was the second most important blood culture isolate after *Streptococcus pneumoniae* in children with invasive bacterial disease [1]. Similarly, a study in an urban hospital of The Gambia revealed that NTS represented 8.6% of the bacteraemia cases [2]. NTS also cause serious dysentery and septicaemia particularly in young infants [3], [4]. However, little is known about environmental reservoirs and predominant modes of transmission especially in the African context [5], [6]. Various sources including farm animals, pets and reptiles have been potentially implicated in the transmission of NTS between animals and humans but their direct involvement in the transmission has never been demonstrated [7]. The asymptomatic *Salmonella* carrier state in poultry has serious consequences on food safety and public health due to the risks of food poisoning following consumption of contaminated products**.** *Salmonella* enterica serovar Enteritidis can persist in the caecum or ovaries of chickens without triggering any clinical signs. Salmonellosis in young chickens may cause high mortality as a result of severe diarrhoea and dehydration, and include a greater risk of evolving into a carrier state in the surviving animals [8], [9], [10]. Small ruminants, such as sheep and goats, are also potential carriers of *Salmonella* [11], [12]. In The Gambia, like in most of the African countries, the majority of the population lives in rural areas and depends on agriculture and livestock. In these areas, the most common animals kept in the compound are chickens, sheep and goats. Therefore, farmers and their families often live in close contact with their livestock and even under the same roof and are thus at increased risk of contracting zoonotic infections, particularly children who often play on the ground. Domestic animals may thus play an important role in the maintenance and transmission of NTS to humans at the community level. Investigating the relatedness between human and animal strains of NTS could provide useful information about the epidemiology of these pathogens. Our study seeks to assess the contribution of domestic animals to the transmission of NTS to humans and to study the genetic relatedness between human and animal strains. This study will also provide useful information about the prevalence of NTS in domestic animals in rural areas of The Gambia.

## Materials and Methods

### Ethics statement

The study protocol and consent form were approved by the Ethics Committees of the Joint Gambian Government/MRC Ethics Committee and the Ethics Committees of the London School of Hygiene and Tropical Medicine, UK and by the Institutional Review Board of the University of Maryland, Baltimore. For any patient eligible for the study written informed consent was obtained prior to their enrollment after the objectives and risks and benefits associated with participation. 95% of eligible subjects agreed to participate. If the participant was illiterate, a witness who was present throughout the consent procedure completed the necessary portions and signed the consent form; the parent/participant marked the consent form (either fingerprint or other notation) cases. If the person was literate, then he/she read and signed the consent form.

All animals used in this study were handled by professional veterinary staff in strict accordance with good animal practice as defined by the Gambian Government/ITC code of practice for the care and use of animals for scientific purposes. All animal work were conducted with ethical approval from both the Joint Gambian Government/ MRC Ethics Committee and the Ethics Committees of the London School of Hygiene and Tropical Medicine, UK.

Animal owners gave their written informed consent to examine and take rectal swabs from their animals and gave permission to publish questionnaire results from this study.

### Study design and sample collection

**Figure 1:**
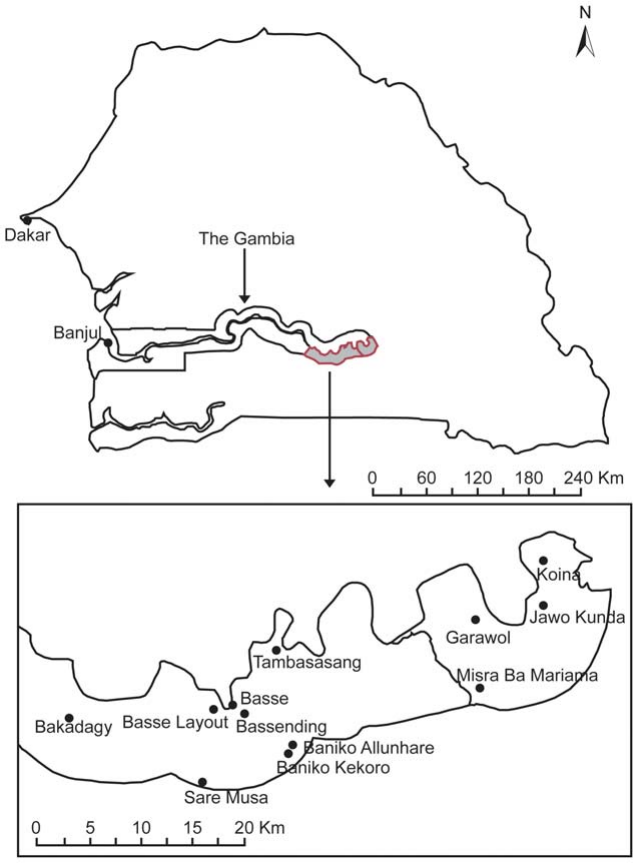
Map of the Gambia showing the locations where Non-Typhoidal *Salmonella* were recovered in humans from the surveillance area in The Gambia.

Human NTS was obtained through active population-based case-control surveillance between December 2007 and February 2009 which was designed to determine the aetiology and epidemiology of enteric infections in Gambian children less than five years of age as part of the Gates Enteric Multicentre Study (GEMS). The entire surveillance area (figure 1) including all the compounds was mapped under GPS coordinates. This surveillance area represented a total population of 152,393 of which 27,567 are less than five years of age. Children under five years of age who presented with severe diarrhoea (i.e., diarrhoea with dehydration, dysentery, or requiring hospitalization) within 3 days of onset of diarrhea were eligible to participate. For each enrolled child with diarrhoea, one healthy control child without diarrhoea was randomly selected from the community in which the case resided, matched to the case by age, gender, and time of presentation. After providing informed consent from the parent/guardian of each case or control a single, fresh, whole stool specimen was collected from cases and controls and cultured to detect bacteria species (*Aeromonas* spp., *Campylobacter* spp, *Salmonella Typhi*, NTS, *Shigella* spp, *Vibrio* spp, diarrheagenic *E. coli* strains), viral (Rotavirus, Adenovirus, Astrovirus, Norovirus, Sapovirus,) and protozoa (*Cryptosporidium* spp *Entamoeba histolytica*, *Giardia lamblia*).

During the surveillance period, 495 diarrhoea cases were identified and NTS were isolated from eight patients and six from community healthy controls. The 14 children were enrolled in this study. and traced back to their family compounds and five apparently healthy animals per species (chicken, sheep and goat) residing in the same household as the child were randomly enrolled within a week of isolating NTS from humans.

Anal swabs were collected from 210 household contact animals and 21 NTS strains were isolated from the faeces.

### Bacterial analysis

Stool specimens were transported in buffered glycerol saline (BGS) to the laboratory and processed within 6 hours of collection. Stools were plated on Xylose Lactose Desoxycholate (XLD) and MacConkey (MAC) agar and incubated at 36°C for 24 hours. Suspected non lactose fermenter colonies were subjected to biochemical reactions using Analytical Profile Index 20 Enteric (API 20E) according to manufactures' instructions (BioMerieux SA, REF 20 100/20 160). Serotyping was done by slide agglutination using Salmonella polyvalent and monovalent O and H antisera (Diagnostic Pasteur, Paris, France) according to the Kauffmann-White classification scheme [13].

### Antimicrobial susceptibility testing

Antimicrobial susceptibility tests were performed on Muller-Hinton agar (Oxoid, USA) using the agar diffusion method with the Bio-Rad discs (Marne-La-Coquette, France) according to the guidelines of the Antibiogram Committee of the French Society for Microbiology (CA-SFM) [14]. Strains were tested with 22 antimicrobial disks (Bio-Rad): amoxicillin (25 mg), amoxicillin (20 mg) plus clavulanic acid (10 mg), ticarcillin (75 mg), cephalotin (30 mg), cefoxitin (30 mg), cefotaxime (30 mg), ceftazidime (30 mg), tobramycin (10 mg), amikacin (30 mg), nalidixic acid (30 mg), pefloxacin (5 mg), norfloxacin (10 mg), trimethoprim (1.2 mg) plus sulfamethoxazole (23.75 mg), tetracycline (30 mg), chloramphenicol (30 mg), gentamicin (10 mg), trimethoprim (300 mg), ciprofloxacin (5 mg), spectinomycin (100 mg), streptomycin (10 mg), sulfonamides (200 mg), and nitrofurantoin (300 mg). Diameters of the inhibition zones were measured with OSIRIS version 3.6xE2.0 (Bio-Rad), and results were interpreted as susceptible, intermediate, or resistant according to the recommendations of the CA-SFM [14]. All these tests were carried out at the MRC microbiology laboratory which is enrolled in the external quality assurance programme of the United Kingdom National External Quality Assessment Scheme [15].

### Multi Locus Sequence Typing (MLST)

MLST was performed on the 35 *Salmonella* isolates as previously described [3]. The seven genes targeted were *aroC*, *dnaN*, *hemD, hisD*, *purE*, *and thrA*. Amplification of all genes was carried out in a 25 µl reaction mixture of the following items: 10xBuffer with 1.5 mm MgCl2 (2.5 µl); 2 mM dNTP'S (0.5 µl); 12.5 mM forward primer (1 µl); 12.5 mM reverse primer (1 µl); 5 U/µl Qiagen Hotstart *Taq* Polymerase (0.25 µl); Template (cell lysate) (2 µl) and 17.75 µl sterile DNA free water. PCR cycling conditions were as follows: 10 min at 94°C, followed by 32 cycles of 94°C for 1 min, 55°C for 1 min and 72°C for 1 min, and a final extension at 72°C for 5 min. 2 µl aliquots of PCR products were separated on 1% agarose gel electrophoresis, and visualized with ethidium bromide staining and UV illumination, and using a gel documentation system. PCR products were purified using Qiagen kit (Qiagen). Sequencing was done on both strands with BigDye Terminator Cycle Sequencing kit (Applied Biosystems, UK). The labelled fragments were separated by size using 3130xl Genetic Analyser (Applied Biosystems, UK). Sequences were edited and complementary sense antisense fragments were aligned using the Laser Gene DNA star 7.1 software. Finally, the sequences were submitted to the MLST database website [16] and assigned to existing or novel allele or sequence type numbers defined by the database.

### Statistical, mapping and cluster analysis

**Figure 2:**
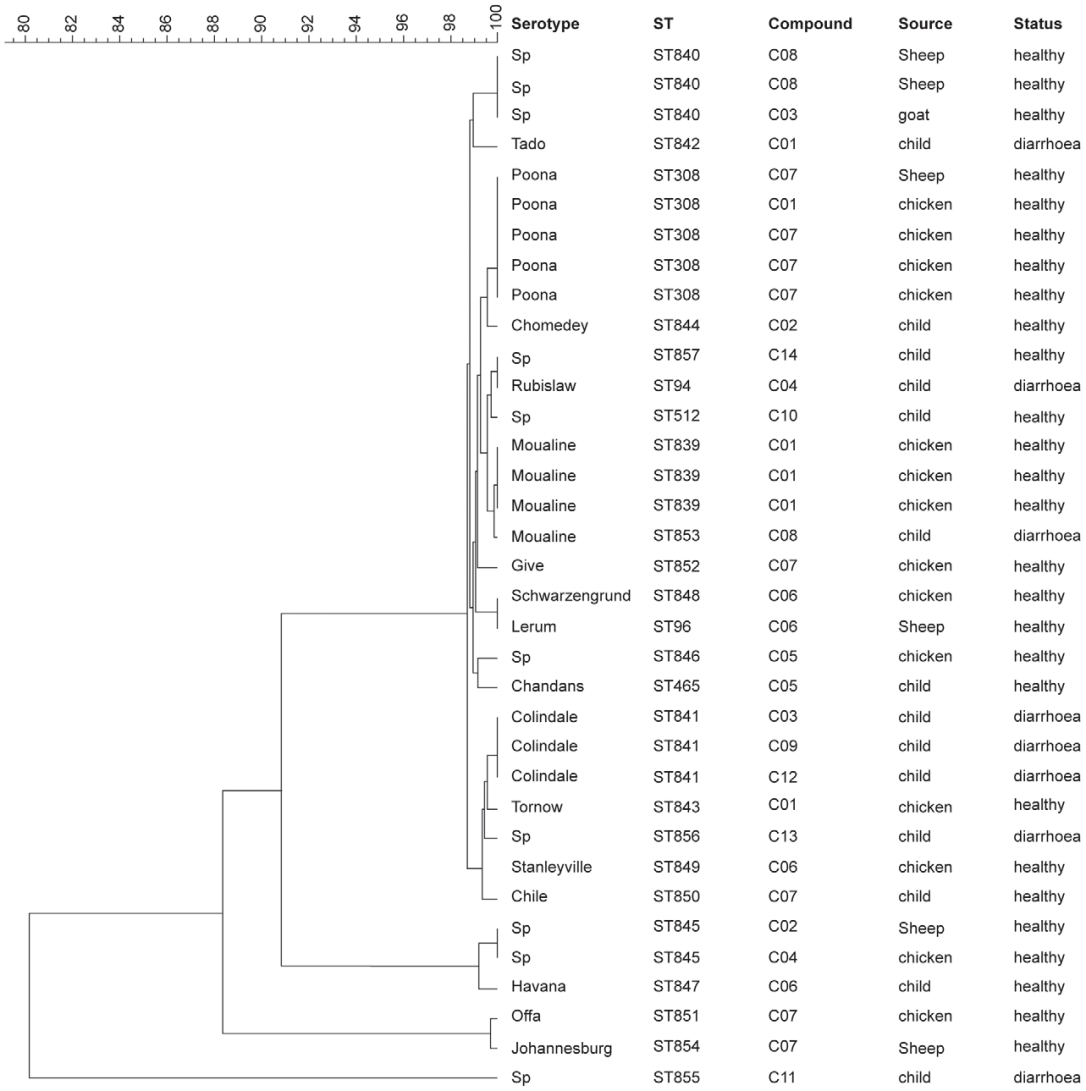
Dendrogram based on the concatenated sequences of the seven alleles from the 35 NTS. The scale bar represents percentage (%) of similarity.

Tests of association were done using Fisher's exact test in Stata 11 (StataCorp. 2009. Stata Statistical Software: Release 11. College Station, TX: StataCorp LP). Stata provides one-sided p-values only for Fisher's exact test unless the table is 2×2 and results with p-values of less than 0.05 for the one-sided test were considered statistically significant. The parameters were grouped for the purpose of the analysis. The secondary diagnosis was categorized into three groups for the Fisher's exact test: group 1, children co-diagnosed with another disease other than malaria; group 2, children co-diagnosed with malaria and group 3, children who were not co-diagnosed with any other disease. The age was also categorized into 2 groups: less than or equal to 18 months (the average age), and more than 18 months. The mapping of the case locations was done using Arc Gis 9.3 software. To perform the cluster analysis of the serovars, MLST data were analysed with Bionumerics software (version 4.0; Applied Maths, Sint-Martens-Latem, Belgium). Analysis using a hierarchic unweighed pair group method (UPGMA) with averaging was used to generate a dendrogram describing the relationship among *Salmonella* serovars (figure 2).

## Results

### Phenotypic diversity of *Salmonella* serovars in the human and animal population

A diversity of serovars was found in both the human and animal population. None of the compounds showed similar serovars in both humans and animals (table 1). Nevertheless, one serovar namely *S*. Moualine was simultaneously found in a diarrheic child and in a chicken but in different compounds: C1 in Banico Allunhare and C8 in Koina (table 1, figure 1). MLST revealed a single locus variant at *sucA* between those 2 strains (table 1). In compounds 9 (C09), 10 (C10), 11 (C11), 12 (C12), 13 (C13) and 14 (C14), no *Salmonella* was isolated from animals (Table 1). The most prevalent serovars were *S*. Colindale in the human population (21.42%) and *S*. Poona in the animal population (14.28%).

**Table 1:** *Salmonella* strains isolated from humans and animals in the same compound and their Sequence Types profiles.

										MLST allelic profile				
Strain ID	Compound	Village	Host	Status	Serovar	Antigenic formula	*aroC*	*dnaN*	*hemD*	*hisD*	*purE*	*sucA*	*thrA*	ST
100661	C01	Banico allunhare	child	diarrheic	Tado	8,20:c:z6:-	197	70	153	4	90	182	139	842
101	C01		chicken	healthy	Moualine	45:06:00	13	169	8	104	41	23	4	839
102	C01		chicken	healthy	Moualine	45:06:00	13	169	8	104	41	23	4	839
103	C01		chicken	healthy	Moualine	45:6:-	13	169	8	104	41	23	4	839
105	C01		chicken	healthy	Tornow	45:gm:-	156	54	68	49	39	9	14	843
127	C01		chicken	healthy	Poona	13,22:z:1,6:-	22	104	25	113	12	83	4	308
100807	C02	Sabi	child	healthy	Chomedey	8,20:z10:enz15:-	13	11	17	113	59	71	4	844
133	C02		sheep	healthy	sp	OME	42	46	152	239	12	197	4	845
102099	C03	Bassending	child	diarrheic	Colindale	6,7:r:1,7:-	6	7	8	10	39	10	14	841
151	C03		goat	healthy	sp	OME	6	157	44	190	159	170	22	840
100385	C04	Garawol	child	healthy	Rubislaw	11:r:enx:-	42	46	48	239	12	13	4	94
145	C04		chicken	healthy	sp	OME	42	46	152	239	12	197	4	845
100123	C05	Basse	child	healthy	Chandans	11:d:enx:-	83	63	49	154	33	58	137	465
146	C05		chicken	healthy	sp	OME	37	31	35	14	8	6	4	846
100225	C06	Bakadagy	child	healthy	Havana	13,23:g,f:-	106	70	152	132	34	9	168	847
115	C06		chicken	healthy	Schwarzengrund	4,12:d:1,7:-	43	47	25	49	41	15	3	848
119	C06		sheep	healthy	Lerum	1,3,19:z:1,7:-	43	47	49	49	41	15	3	96
121	C06		chicken	healthy	Stanleyville	4,12:HMC	17	7	35	9	2	9	14	849
100400	C07	Misra ba mariama	child	Healthy	Chile	6,7:z:1,2:-	121	7	10	103	2	10	14	850
109	C07		chicken	healthy	Offa	41:z38:-	111	104	17	256	226	197	65	851
110	C07		chicken	healthy	Give	3,10:l,v:z26:-	42	11	17	42	40	71	102	852
137	C07		chicken	healthy	Poona	13,22:z:1,6:-	22	104	25	113	12	83	4	308
143	C07		chicken	healthy	Poona	13,22:z:1,6:	22	104	25	113	12	83	4	308
144	C07		chicken	healthy	Poona	13,22:z:1,6:	22	104	25	113	12	83	4	308
107	C07		sheep	healthy	Poona	13,22:z:1,6:	22	104	25	113	12	83	4	308
141	C07		sheep	healthy	Johannesburg	40:b:enx:-	111	11	17	256	12	197	65	854
100887	C08	Koina	child	diarrheic	Moualine	45:6:-	13	169	8	104	41	13	4	853
147	C08		sheep	healthy	sp	OME	6	157	44	190	159	170	22	840
149	C08		sheep	healthy	sp	OME	6	157	44	190	159	170	22	840
100025	C09	Sare musa	child	diarrheic	Colindale	6,7:r:1,7:-	6	7	8	10	39	10	14	841
100054	C10	Jawo kunda	child	healthy	sp	OME	13	109	49	13	12	13	4	512
100132	C11	Baniko kekoro	child	diarrheic	sp	OME	61	112	8	10	134	137	4	855
100288	C12	Garawol	child	diarrheic	Colindale	6,7:r:1,7:-	6	7	8	10	39	10	14	841
102044	C13	Basse layout	child	diarrheic	sp	OME	70	7	10	10	2	142	14	856
100392	C14	Tambasasang	child	diarrheic	sp	OME	42	36	48	239	12	13	4	857

The proportion of *Salmonella* isolated was higher in the chicken population than in other species: 66.6%, 28.6% and 4.8% in chickens, sheep and goats, respectively. In the same compound, a diversity of *Salmonella* serovars were circulating at animal level especially in chickens; the number varying from 2 to 3 different serovars: in compound 1 (C1), *S.* Moualine, *S.* Tornow and *S.* Poona; in compound 6 (C6), *S.* Schwarzengrund and *S.* Stanleyville and in compound 7 (C7), *S.* Offa, *S.* Give and *S.* Poona (table 1).

### Epidemiological aspects of salmonellosis in children

The mean age of children enrolled in the study was (to the nearest integer) 18 months and the age varied between 9 and 26 months (table 2). All children who presented with diarrhoea except one were secondarily diagnosed with another disease such as malaria (4 children) or other disease symptoms including fever, or cough (3 children) (table 2). There was a significant association (p-value<0.01) between expressing clinical signs of salmonellosis, i.e. diarrhoea and being co-diagnosed with a secondary disease (table 3). Age was not associated with the expression of clinical signs of salmonellosis (p-value = 0.16).

**Table 2:** Clinical symptoms present in 14 Gambian children with Non-Typhoidal *Salmonella* infections.

ID	age (months)	first diagnosis	second diagnosis
100025	26	diarrhoea	none
100132	22	diarrhoea	malaria
102044	26	diarrhoea	malaria
100288	17	diarrhoea	malaria
100661	20	diarrhoea	cough, fever more than 38°C
100385	9	diarrhoea	malaria
100887	22	diarrhoea	cough with difficult breathing
102099	26	diarrhoea	belly pain, fever more than 38°C
100392	10	Healthy	none
100123	20	Healthy	none
100225	9	Healthy	none
100807	21	Healthy	none
100400	9	Healthy	none
100054	10	Healthy	none

**Table 3:** Categorisation of the patient population by diagnostic criteria.

diagnosis 1	diagnosis 2			
	other disease symptoms	malaria	none	total
healthy	0	0+	6	6
diarrhoea	3	4	1	8
Total	3	4	7	14

### Drug susceptibility

All serovars were fully susceptible to all antibiotics tested except one of each the following serovars: *Salmonella* enteritica serovar Poona, *Salmonella* enteritica serovar Johannesburg, *Salmonella* enteritica serovar Chile and *Salmonella* enteritica serovar Colindale which were resistant to streptomycin.

### Genetic diversity of *Salmonella* serovars using Multilocus Sequence Typing

Four new alleles were discovered: *hemD* (152), *hemD* (153), *hisD* (256) and *purE* (226) and eighteen novel sequence types (ST) (table 1). Similar serovars exhibited the same allelic profiles, except *S.* Moualine which had two different allelic profiles for the serovars isolated from humans and animals (table 1).

The seven housekeeping genes were concatenated for all isolates and the UPGMA tree was constructed (figure 2). All *Salmonella* genotypes had at least 80% similarity and the majority varied between 99% and 100%. *Salmonella* genotypes causing diarrhoea in children were always clustered with animal genotypes. Both *Salmonella* enteritica serovar Moualine isolated from a chicken and a child was clustered (figure 2).

## Discussion

### *Salmonella* serovars are phenotypically diverse, genetically close but remain clonally different between humans and domestic animal sources

The serovar diversity of NTS within the human and animal population was high showing that *Salmonella* is carried by both humans and animals in the community. The serovars were also widely geographically distributed in our surveillance area located in a typical African rural setting in The Gambia. We showed that several *Salmonella* serovars were circulating in the chicken population within the same compound or household; such as compounds 1 (C1), 6 (C6) and 7 (C7) respectively in the following 3 villages: Baniko Allunhare, Bagadagy and Misra Ba Mariama (figure 1). The higher proportion of *Salmonella* serovars in the chicken population compared to the goats and sheep is not surprising as it is known that chicken is the most important reservoir of NTS and thus thought to be the major source of transmission to humans [17]. *Salmonella* can persist in the chicken cecum or ovaries without triggering clinical signs in the host. Salmonellosis in young chickens may cause high mortality as a result of severe diarrhoea and dehydration, and creates a greater risk of evolving into a carrier state in the animals which survive [8], [9], [10]. The asymptomatic *Salmonella* carrier state in poultry has serious consequences for food safety and public health due to the risks of food poisoning following consumption of contaminated products**.** Small ruminants, such as sheep and goats, are also potential carriers of *Salmonella* [11], [12]. In Ethiopia, a study indicated that *Salmonella* is common in apparently healthy slaughtered sheep and goats. It also showed the presence of a wide range of *Salmonella* serovars in sheep and goats, which are of veterinary and public health significance [18]. The high rate of NTS clones circulating in the same compound could lead to mixed infection or carriage within the chicken population. This situation could result in extensive genetic diversity and variability due to frequent intraspecific recombination as it occurs with *Helicobacter pylori* [19]. This could have as consequence a wider range of clones and thus more difficulties to control NTS infections at animal level.

The diversity of serovars that we observed in this study is different from what we previously reported [3] where *Salmonella enterica* serovar Enteritidis was the most common serovar (80.6%) followed by *Salmonella enterica* serovar Typhimurium (8.0%) among NTS isolated from children with pneumonia and/or septicemia patients. It appears that the epidemiology of NTS is changing in The Gambia or the serovars detected might be site or disease specific, i.e. gastroenteritis vs. systemic infections.

MLST provides the best phylogenetic-relationship inference for the *Salmonella* genus [20]. Therefore, it may be invaluable for determination of the relationship among various *Salmonella* strains and serovars [21] **.** As expected, the similarity matrix (figure 2) of the serovars revealed close genetic relationship (>80% and the majority between 99 to 100%) between human and animal serovars, showing the genetic homogeneity of *Salmonella.* A study done in Senegal has also revealed a high degree of similarity among *Salmonella* enteritica serovar Brancaster and *Salmonella* enteritica serovar Enteritidis serovars from poultry and from humans by the use of PFGE techniques [22], but direct evidence of *Salmonella* transmission from poultry to humans could not be provided.

The high degree of similarity between human and animal serovars supports the theory that *Salmonella* clones are stable [23]. The genetic tree has also revealed that all lineages contained isolates of mixed origin (human and animal). From the present data, there is therefore no indication of clonal groups or lineages that are adapted to any specific host. These findings support the conclusions of other authors who used the same techniques (MLST) with *Salmonella* from human and veterinary sources in Denmark [24] or a different technique like Pulsed Field Gel Electrophoresis (PFGE) with isolates from humans and animals, food or the environment in close contact with humans, which was the case in Kenya [25]. Like in the Kenya study [25], we also showed that there was no relatedness between NTS genotypes from humans and those from animals in close contact to humans, this other potential sources of transmission such as environmental or the human-to-human transmission need to be examined.

### Expression of clinical signs of salmonellosis is associated with the existence of a secondary disease especially malaria

A statistical significant association (P<0.05) was observed between children expressing clinical signs of salmonellosis (diarrhoea) and co-diagnosis with malaria (table 3). The association between salmonellosis and immunocompromising diseases is well known in the African context. NTS infections are usually associated with opportunistic infections especially in immuno-compromised patients, *e.g.* HIV-infected adults [26], [27] or with other diseases like malaria or anemia [1], [5], [28], [29]. Children especially those less than 3 years old are the age-group at risk of expressing clinical signs of salmonellosis [1], [26] **.** The contradiction between our observation and those of most other authors is certainly due to the small sample size and the small range of age groups in our study. All individuals were in fact less than three years old. Malaria has long been suspected to increase the risk of invasive NTS infection and might contribute to the seasonality of NTS disease, although the mechanism underlying the association between malaria and NTS is only partially understood [6].

### Animal and human NTS serovars are fully susceptible to commonly used antibiotics in The Gambia

All serovars were susceptible to most commonly used antibiotics for the treatment of clinical infections in The Gambia such as amoxicillin, amoxicillin plus clavulanic acid, trimethoprim plus sulfamethoxazole, tetracycline, streptomycin and chloramphenicol and also to cephalosporins of the third generation which are considered as the drugs of choice for invasive *Salmonella* infections in humans. This result is in contrast to previous studies done in urban [1], [2] and rural [3] areas in The Gambia where *Salmonella* strains expressed multi-resistance to several commonly used antibiotics. This could be explained in part by the fact that the NTS isolated from those studies were from invasive cases (pneumonia and sepsis) whereas this study focused on non-invasive cases (diarrhea). In addition, in those studies *Salmonella enterica* serovar Enteritidis was the most common serovar followed by *Salmonella enterica* serovar Typhimurium. While these serovars were not detected in this study as a result of temporal trends of childhood NTS infection in The Gambia {Mackenzie, 2010 #33}. There is lack of resistance of serovars to antibiotics in rural areas even to those commonly used in the hospitals to treat bacterial infections. This is due to the fact that in rural areas, NTS infections are not treated because patients are often not conducted to the hospital due to poor access to medical centers, lack of transport facilities and of financial resources. Our findings suggest that these drugs remain suitable for the treatment of salmonellosis in humans and animals. However, we have to interpret these results with caution because the sample size in our study was small.

Our study showed that the use of serotyping data combined with MLST and phylogenetic analysis can provide important information about the epidemiology of NTS in humans and animals. However, our results do not support the hypothesis that humans and animals in close contact in the same household carry genotypically similar *Salmonella* serovars. Nevertheless these findings have stirred up the problem of the transmission of NTS in African context and suggest that poultry may play an important part in the epidemiology of Salmonella infections of this condition. A better control of malaria may lead to a reduction in the incidence of invasive NTS disease in The Gambia. Multidrug resistance has not yet been a problem in human and animal NTS isolates in this area of the country. Thus, commonly available drugs may still be used for the treatment of NTS infections in rural Gambia. Nevertheless, public authorities must be alert to detect any change in the behavior of *Salmonella* towards antibiotics, with a view of establishing appropriate control measures for use of these drugs in humans and animals.
